# Crystal structure and Hirshfeld surface analysis of (*Z*)-4-{[4-(3-methyl-3-phenyl­cyclo­but­yl)thia­zol-2-yl]amino}-4-oxobut-2-enoic acid

**DOI:** 10.1107/S2056989022000032

**Published:** 2022-01-07

**Authors:** Okan Simsek, Muharrem Dincer, Necmi Dege, Eiad Saif, Ibrahim Yilmaz, Alaaddin Cukurovali

**Affiliations:** a Department of Physics, Faculty of Arts and Sciences, Ondokuz Mayıs University, 55139, Samsun, Turkey; b Department of Computer and Electronic Engineering Technology, Sanaa Community College, Sanaa, Yemen; c Ondokuz Mayıs University, Faculty of Engineering, Department of Electrical and Electronic Engineering, 55139, Samsun, Turkey; d Department of Chemistry, Kamil Ozdag Science, Karamanoğlu Mehmetbey University, 70200, Karaman, Turkey; e Department of Chemistry, Sciences Faculty, Fırat University, 23119, Elazığ, Turkey

**Keywords:** crystal structure, cyclo­but­yl, thia­zole, 4-oxobut-2-enoic acid, Hirshfeld surface analysis

## Abstract

The title cyclo­butyl compound, C_18_H_18_N_2_O_3_S, was synthesized by the inter­action of 4-(3-methyl-3-phenyl­cyclo­but­yl)thia­zol-2-amine and maleic anhydride, and crystallizes in the ortho­rhom­bic space group *P*2_1_2_1_2_1_ with *Z*′ = 1. The mol­ecular geometry is partially stabilized by an intra­molecular N—H⋯O hydrogen bond forming an *S*_1_^1^(7) ring motif. The mol­ecule is non-planar with a dihedral angle of 88.29 (11)° between the thia­zole and benzene rings. In the crystal, the mol­ecules are linked by O—H⋯N hydrogen bonds, forming supra­molecular ribbons with *C*_1_^1^(9) chain motifs. To further analyze the inter­molecular inter­actions, a Hirshfeld surface analysis was performed. The results indicate that the most important contributions to the overall surface are from H⋯H (43%), C⋯H (18%), O⋯H (17%) and N⋯H (6%), inter­actions.

## Abstract 2

The title compound is a cyclo­butyl compound that adopts a *Z* configuration. The mol­ecular structure is stabilized by an N—H⋯O hydrogen bond, forming an 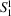 (7) ring motif. In the crystal, mol­ecules are linked by pairs of O—H⋯N hydrogen bonds, forming supra­molecular ribbons linked *via C*_1_^1^(9) ring motifs.

## Chemical context

Cyclo­butanes are four-membered carbocycles, which present a unique structural feature in bioactive natural products. Many natural cyclo­butanes contain various substituents (Hui *et al.*, 2021 ▸). Complex derivatives of cyclo­butanes have an important place in biology and biotechnology (Dinçer *et al.*, 2004 ▸). In addition, it has been shown that 3-substituted cyclo­butane carb­oxy­lic acid derivatives exhibit anti-inflammatory and anti­depressant activities (Dehmlow & Schmidt, 1990 ▸), and can also form liquid crystals (Coghi *et al.*, 1976 ▸). In addition, thia­zole is a heterocyclic organic compound that has a five-membered ring containing three carbon, one sulfur, and one nitro­gen atoms. Thia­zoles are found in many potent biologically active compounds, such as sulfa­thia­zole (anti­microbial drug), ritonavir (anti­retroviral drug), abafungin (anti­fungal drug), bleomycine, and tiazofurin (anti­neoplastic drug) (Kashyap *et al.*, 2012 ▸; Mohapatra *et al.*, 2019 ▸). In this study, (*Z*)-4-{[4-(3-methyl-3-phenyl­cyclo­but­yl)thia­zol-2-yl]amino}-4-oxobut-2-enoic acid was synthesized from 4-(3-methyl-3-phenyl­cyclo­but­yl)thia­zol-2-amine and maleic anhydride and was characterized by single crystal X-ray diffraction and the crystal packing was analyzed using Hirshfeld surface analysis.

## Structural commentary

**Figure 1:**
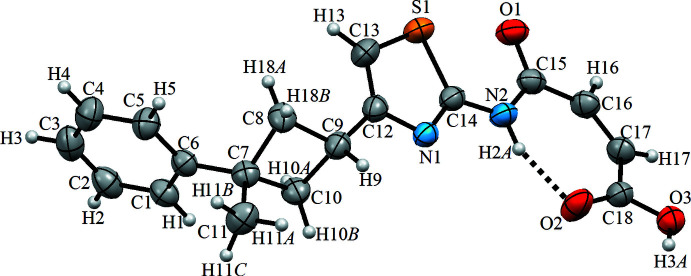
The mol­ecular structure of the title compound with displacement ellipsoids drawn at the 40% probability level. Dashed lines denote the intra­molecular N—H⋯O hydrogen bonds forming an 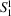 (7) ring motif.

The title cyclo­butyl derivative crystallizes in the ortho­rhom­bic *P*2_1_2_1_2_1_ space group with *Z*′ = 1. Its mol­ecular structure is illustrated in Fig. 1 ▸, showing the intra­molecular N—H⋯O hydrogen bond forming an 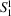 (7) ring motif. The mol­ecule is non-planar as the thia­zole and benzene rings are twisted with respect to each other, subtending a dihedral angle of 88.29 (11)°. In addition, the cyclo­butyl ring is twisted by 58.1 (2) and 40.2 (2)°, with respect to the thia­zole, and benzene rings. In the thia­zole ring, the C12—N1 bond length is 1.386 (4) Å and classified as a single bond. 
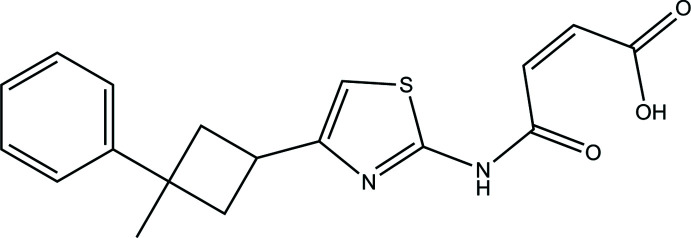


The cyclo­butane adopts a puckered (butterfly) conformation. The average carbon–carbon (C—C) bond lengths within the ring is 1.5506 Å, with average C—C—C bond angles of 88.89°, while the average torsion angle within the C_4_ ring is 15.83°. When these parameters are compared with the recently published cyclo­butane derivatives (Gumus *et al.*, 2021 ▸), it is seen that there are no considerable differences. The S1—C13 and S1—C14 bond lengths are 1.727 (4) and 1.716 (3) Å, typical of a single bond. These values are comparable to those reported previously [1.718 (4) Å (Kansiz *et al.*, 2021 ▸) and 1.727 (9) Å (Qadir *et al.*, 2021 ▸)], but are longer than the values of 1.685 (4) and 1.698 (3) Å reported by Albayati *et al.* (2020 ▸).

## Supra­molecular features

**Figure 2:**
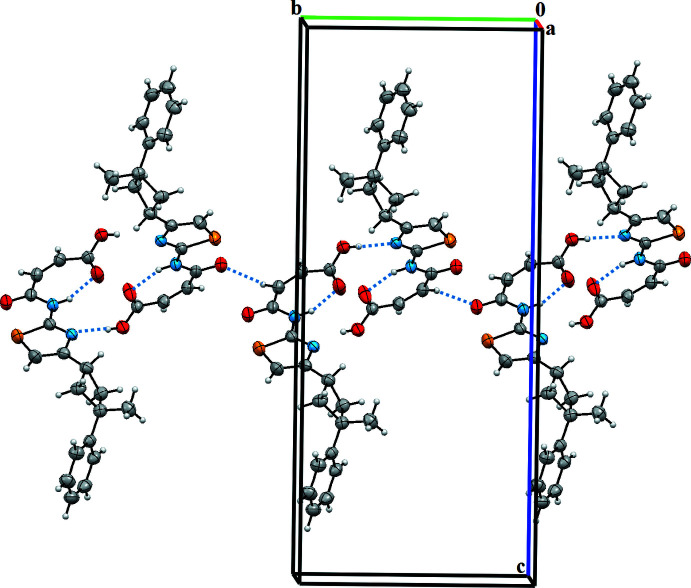
A view of the crystal packing of the title compound. Blue dashed lines denote the inter­molecular O3—H3*A*⋯N1 hydrogen bonds forming a 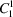 (9) motif (Table 1 ▸).

The crystal packing of the title compound (Fig. 2 ▸) features inter­molecular hydrogen bonds (C16—H16⋯O1^i^ and O3—H3*A*⋯N1^ii^; symmetry codes are given in Table 1 ▸). In the crystal, the mol­ecules are linked by O3—H3*A*⋯N1 hydrogen bonds forming supra­molecular ribbons *via C*_1_^1^(9) motifs. Adjacent ribbons are connected by C16—H16⋯O1 hydrogen bonds, leading to the formation of layers lying parallel to the *bc* plane.

## Database survey

A search of the Cambridge Structural Database (CSD Version 5.42, update of September 2021; Groom *et al.*, 2016 ▸) for the 4-(3-methyl-3-phenyl­cyclo­but­yl)thia­zole moiety gave several hits including 4-{[4-(3-mesityl-3-methyl­cyclo­but­yl)-1,3-thia­zol-2-yl]amino}-4-oxo­butanoic acid dihydrate (CIBQIP; Şen *et al.*, 2013 ▸), 2-[4-(3-(2,5-di­methyl­phen­yl)-3-methyl­cyclo­but­yl]-1,3-thia­zol-2-yl)-1*H*-iso­indole-1,3(2*H*)-dione (HAMKAJ; Öz­demir *et al.*, 2010 ▸), 2-chloro-*N*-[4-(3-methyl-3-phenyl­cyclo­but­yl)-1,3-thia­zol-2-yl]-*N*′-(naphthalen-1-yl­methyl­idene)ace­to­hydrazide (IJULIJ; Inkaya *et al.*, 2011*a* ▸), *N*-[4-(3-methyl-3-phenyl­cyclo­but­yl)-1,3-thia­zol-2-yl]acetamide (LUXDIU; Ekici *et al.*, 2020 ▸), *N*′-benzyl­idene-2-chloro-*N*-[4-(3-methyl-3-phenyl­cyclo­but­yl)-1,3-thia­zol-2-yl]acetohydrazide (PICZUY; Demir *et al.*, 2012 ▸), 2-chloro-*N*′-[4-(di­methyl­amino)­benzyl­idene]-*N*-[4-(3-methyl-3-phenyl­cyclo­but­yl)-1,3-thia­zol-2-yl]acetohydrazide (QAKFUF; Inkaya *et al.*, 2011*b* ▸), 2-chloro-*N*′-(2-furyl­methyl­ene)-*N*-[4-(3-methyl-3-phenyl­cyclo­but­yl)-1,3-thia­zol-2-yl]acetohydrazide (URECEB; Demir *et al.*, 2016 ▸) and 4-[4-(3-mesityl-3-methyl­cyclo­but­yl)-1,3-thia­zol-2-yl]-1-thia-4-aza­spiro­[4.5]decan-3-one (VOXBER; Şen *et al.*, 2015 ▸). In LUXDIU (Ekici *et al.*, 2020 ▸), the cyclo­butyl ring has puckering parameters *Q* = 0.240 (4) Å and θ = 17.67 (2)°, that are close to those for the title compound [*Q* = 0.216 (2) Å and θ = 15.83 (5)°]. The cyclo­butane ring is puckered, with a dihedral angle of 25.20 (5)° in IJULIJ (Inkaya *et al.*, 2011*a* ▸) and 22.99 (47)° in QAKFUF (Inkaya *et al.*, 2011*b* ▸). In HAMKAJ (Özdemir *et al.*, 2010 ▸), the cyclo­butane ring has a puckered conformation with 28.84 (22)°. This value is significantly bigger than those in the literature; 20.03 (3)° (PICZUY; Demir *et al.*, 2012 ▸) and 18.9 (3)° (CIBQIP; Şen *et al.*, 2013 ▸). In the title compound, the C—S bond lengths within the thia­zole ring are 1.727 (4) and 1.716 (3) Å, which are congruent with similar examples from the literature, 1.697 (6) and 1.739 (6) Å (VOXBER; Şen *et al.*, 2015 ▸) and 1.701 (4) and 1.726 (2) Å (URECEB; Demir *et al.*, 2016 ▸). These values are shorter than the standard value for a *Csp* ^2^—S single bond (1.76 Å). In all structures, the phenyl and thia­zole rings are *cis*-related with respect to the cyclo­butane ring. The asymmetric units in all above-mentioned examples contain only one mol­ecule.

## Hirshfeld surface analysis

**Figure 3:**
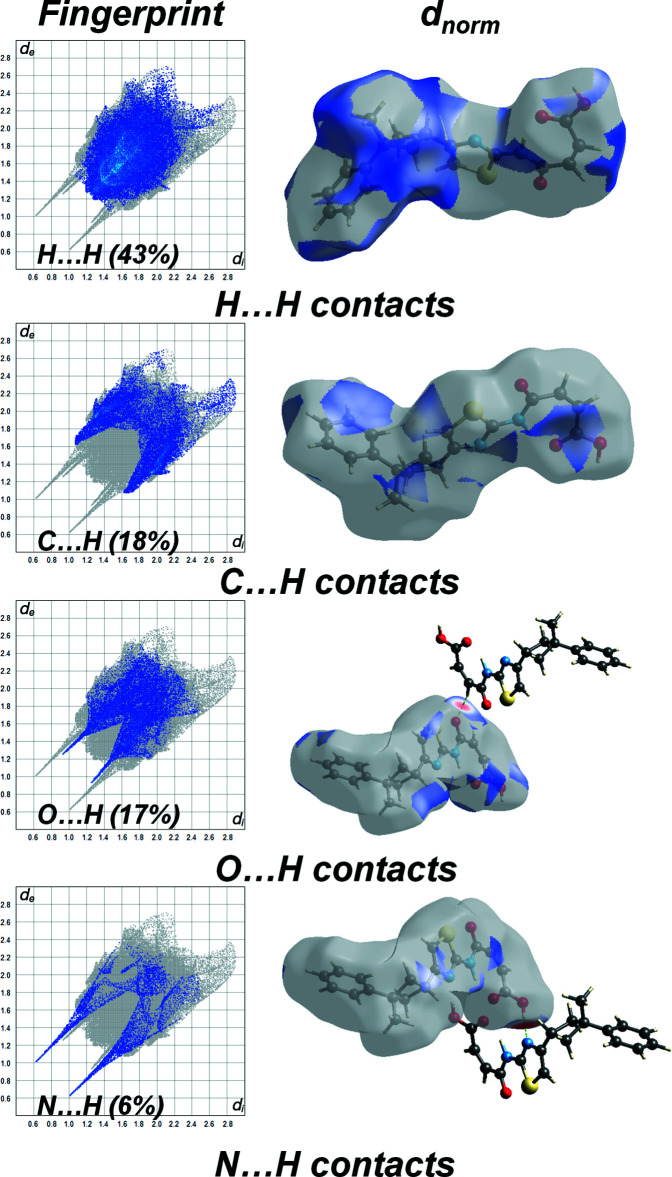
The Hirshfeld surface analysis of the title compound mapped with *d*_norm_ over the range −0.763 to 1.558 showing C—H⋯O and O—H⋯N hydrogen-bonded contacts.

**Figure 4:**
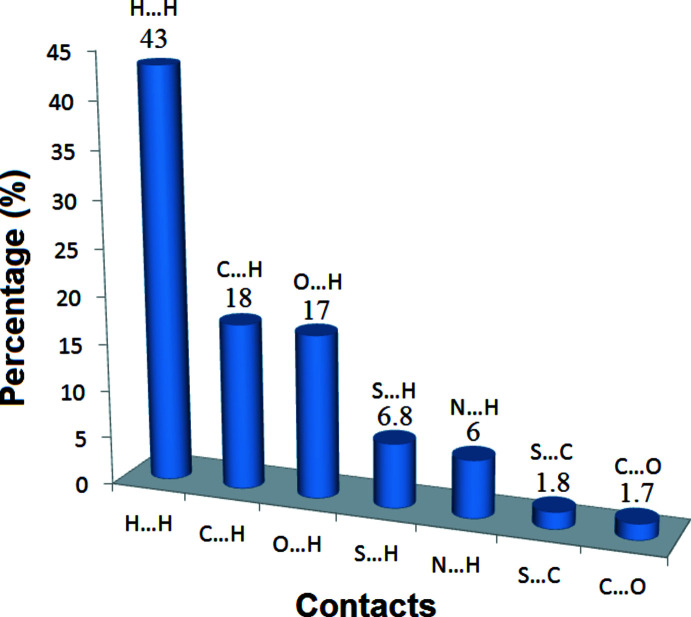
Inter­molecular inter­actions and their percentages in C_18_H_18_N_2_O_3_S.

To compare qu­anti­tatively the different inter­molecular inter­actions affecting the mol­ecular packing in the studied compound, the Hirshfeld surface analysis was employed. The strength of the present inter­molecular inter­actions can be displayed on the Hirshfeld surface (Spackman & Jayatilaka, 2009 ▸) generated by *CrystalExplorer17* (Turner *et al.*, 2017 ▸), here indicated by the red spots (Fig. 3 ▸). Furthermore, the Hirshfeld surface analysis is a valuable tool for predicting the properties of a crystal and its potential applications (Al-thamili *et al.*, 2020 ▸; Ilmi *et al.*, 2020 ▸). The contributions of the different types of inter­molecular inter­actions for the title compound are shown in the two-dimensional fingerprint plots in Fig. 3 ▸. Fig. 4 ▸ displays the diverse contacts and their percentages observed in the crystal structure of the C_18_H_18_O_3_S compound based on the Hirshfeld calculations. The mol­ecular packing of the title compound is mainly controlled by relatively strong O⋯H (17%) and N⋯H (6%) inter­actions ions and by abundant, but weaker, H⋯H (43%) and C⋯H (18%) 8%) van der Waals type interactions. S⋯H (6.8%), S⋯C (1.8%), C⋯O (1.7%), C⋯C (1.7%) and C⋯N (1.5%) contacts are also present. The corresponding fingerprint plots and decomposed *d*_norm_ maps for these inter­actions are shown in Fig. 3 ▸. The results also indicate the presence of N—H⋯O, C—H⋯O and O—H⋯N hydrogen bonds.

## Synthesis and crystallization

**Figure 5:**
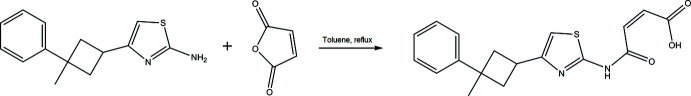
The synthesis of (*Z*)-4-{[4-(3-methyl-3-phenyl­cyclo­but­yl)thia­zol-2-yl]amino}-4-oxobut-2-enoic acid.

A mixture of 4-(3-methyl-3-phenyl­cyclo­but­yl)thia­zol-2-amine (2.4436 g, 10 mmol) and maleic anhydride (0.9806 g, 10 mmol) in 20 mL of dry toluene under argon atmosphere was refluxed for 12 h (monitored by TLC). Solvent was removed under reduced pressure and the residue crystallized from ethanol in the form of brilliant yellow crystals. The reaction scheme is shown in Fig. 5 ▸. Yield 94%, m.p. 460 K. Characteristic IR bands (cm^−1^): 2975–2855 ν(C—H aliphatics), 1670 ν(C=O), 1626 ν(C=O), 1569 ν(C=N azomethine), 699 ν(C—S—C). Characteristic ^1^H NMR shifts (THF-*d*_8_ + acetone-*d*_6_, TMS, ppm): 1.26 (*s*, 3H, –CH_3_), 2.15–2.20 (*m*, 2H, –CH_2_– in cyclo­butane ring), 2.31–2.36 (*m*, 2H, –CH_2_– in cyclo­butane ring), 3.48 (quint, *J* = 9.2 Hz, 2H, >CH– in cyclo­butane ring), 6.10 (*d*, *J* = 12.8 Hz, 1H, –CH=), 6.27 (*d*, *J* = 12.4 Hz, 1H, =CH–), 6.50 (*s*, 1H, S—CH=in thia­zole ring), 6.89–6.92 (*m*, 3H, aromatics), 7.00 (*m*, 2H, aromatics). –OH and –NH– protons of this mol­ecule have not been determined in the ^1^H NMR spectrum. Characteristic ^13^C NMR shifts (THF-*d*_8_ + acetone-*d*_6_, TMS, ppm): 165.30, 162.65, 156.96, 154.69, 152.29, 132.00, 130.84, 127.71, 124.81, 124.21, 107.19, 40.25, 38.16, 30.20, 29.35.

## Refinement

Crystal data, data collection and structure refinement details are summarized in Table 2 ▸. Although the acidic protons from the O–H and N–H bonds could be located in the difference-Fourier map, even very strong distance restraints were not sufficient to obtain proper distances between the parent atom and hydrogen. Therefore, both protons were refined in geometrical positions using the corresponding AFIX instructions with O—H = 0.82 Å and *U*_iso_(H) = 1.5*U*_eq_(O), and N—H = 0.86 Å and *U*_iso_(H) = 1.2*U*_eq_(N), respectively. The C-bound H atoms were positioned geometrically (C—H = 0.93, 0.96, 0.97 and 0.98 Å) and refined using a riding model, with *U*_iso_(H) = 1.5*U*_eq_(C) for methyl H atoms and 1.2*U*_eq_(C) for other H atoms.

## Supplementary Material

Crystal structure: contains datablock(s) I. DOI: 10.1107/S2056989022000032/jq2012sup1.cif

Structure factors: contains datablock(s) I. DOI: 10.1107/S2056989022000032/jq2012Isup2.hkl

Supporting information file. DOI: 10.1107/S2056989022000032/jq2012Isup3.cml

CCDC reference: 1953658

Additional supporting information:  crystallographic information; 3D view; checkCIF report

## Figures and Tables

**Table 1:** Hydrogen-bond geometry (Å, °)

*D*—H⋯*A*	*D*—H	H⋯*A*	*D*⋯*A*	*D*—H⋯*A*
C16—H16⋯O1^i^	0.93	2.35	3.233 (4)	159
N2—H2*A*⋯O2	0.86	1.83	2.651 (4)	158
O3—H3*A*⋯N1^ii^	0.82	1.81	2.607 (3)	165

Symmetry codes: (i) x-{\script{1\over 2}}, -y+{\script{5\over 2}}, -z+1; (ii) x-{\script{1\over 2}}, -y+{\script{3\over 2}}, -z+1.

**Table 2:** Experimental details

Crystal data	
Chemical formula	C_18_H_18_N_2_O_3_S
*M*_r_	342.40
Crystal system, space group	Orthorhombic, *P*2_1_2_1_2_1_
Temperature (K)	296
*a*, *b*, *c* (Å)	5.9685 (4), 11.0580 (9), 26.215 (2)
*V* (Å^3^)	1730.2 (2)
*Z*	4
Radiation type	Mo *K*α
μ (mm^−1^)	0.21
Crystal size (mm)	0.78 × 0.71 × 0.59

Data collection	
Diffractometer	Stoe IPDS 2
Absorption correction	Integration (*X-RED32*; Stoe & Cie, 2002 ▸)
*T*_min_, *T*_max_	0.832, 0.899
No. of measured, independent and observed [*I* > 2σ(*I*)] reflections	7546, 3338, 2648
*R*_int_	0.058
(sin θ/λ)_max_ (Å^−1^)	0.617

Refinement	
*R*[*F* ^2^ > 2σ(*F* ^2^)], *wR*(*F* ^2^), *S*	0.045, 0.113, 0.97
No. of reflections	3338
No. of parameters	218
H-atom treatment	H-atom parameters constrained
Δρ_max_, Δρ_min_ (e Å^−3^)	0.20, −0.16
Absolute structure	Flack *x* determined using 907 quotients [(*I* ^+^)−(*I* ^−^)]/[(*I* ^+^)+(*I* ^−^)] (Parsons *et al.*, 2013 ▸)
Absolute structure parameter	−0.05 (8)

Computer programs: *X-AREA* and *X-RED32* (Stoe & Cie, 2002 ▸), *SHELXT2014/5* (Sheldrick, 2015*a* ▸), *SHELXL2017/1* (Sheldrick, 2015*b* ▸), *ORTEP-3 for Windows* and *WinGX* (Farrugia, 2012 ▸) and *PLATON* (Spek, 2020 ▸).

## supplementary crystallographic information

### Crystal data

**Table:**

C_18_H_18_N_2_O_3_S	*D*_x_ = 1.314 Mg m^−^^3^
*M**_r_* = 342.40	Melting point: 460 K
Orthorhombic, *P*2_1_2_1_2_1_	Mo *K*α radiation, λ = 0.71073 Å
*a* = 5.9685 (4) Å	Cell parameters from 7998 reflections
*b* = 11.0580 (9) Å	θ = 1.6–27.3°
*c* = 26.215 (2) Å	µ = 0.21 mm^−^^1^
*V* = 1730.2 (2) Å^3^	*T* = 296 K
*Z* = 4	Prism, light yellow
*F*(000) = 720	0.78 × 0.71 × 0.59 mm

### Data collection

**Table:**

Stoe IPDS 2 diffractometer	3338 independent reflections
Radiation source: sealed X-ray tube, 12 x 0.4 mm long-fine focus	2648 reflections with *I* > 2σ(*I*)
Detector resolution: 6.67 pixels mm^-1^	*R*_int_ = 0.058
rotation method scans	θ_max_ = 26.0°, θ_min_ = 1.6°
Absorption correction: integration (X-RED32; Stoe & Cie, 2002)	*h* = −7→7
*T*_min_ = 0.832, *T*_max_ = 0.899	*k* = −13→12
7546 measured reflections	*l* = −32→30

### Refinement

**Table:**

Refinement on *F*^2^	Hydrogen site location: inferred from neighbouring sites
Least-squares matrix: full	H-atom parameters constrained
*R*[*F*^2^ > 2σ(*F*^2^)] = 0.045	*w* = 1/[σ^2^(*F*_o_^2^) + (0.0679*P*)^2^] where *P* = (*F*_o_^2^ + 2*F*_c_^2^)/3
*wR*(*F*^2^) = 0.113	(Δ/σ)_max_ = 0.001
*S* = 0.97	Δρ_max_ = 0.20 e Å^−^^3^
3338 reflections	Δρ_min_ = −0.16 e Å^−^^3^
218 parameters	Extinction correction: SHELXL2017/1 (Sheldrick 2015b), Fc^*^=kFc[1+0.001xFc^2^λ^3^/sin(2θ)]^-1/4^
0 restraints	Extinction coefficient: 0.014 (3)
Primary atom site location: structure-invariant direct methods	Absolute structure: Flack *x* determined using 907 quotients [(*I*^+^)-(*I*^-^)]/[(*I*^+^)+(*I*^-^)] (Parsons *et al.*, 2013)
Secondary atom site location: difference Fourier map	Absolute structure parameter: −0.05 (8)

### Special details

**Table:**

Geometry. All esds (except the esd in the dihedral angle between two l.s. planes) are estimated using the full covariance matrix. The cell esds are taken into account individually in the estimation of esds in distances, angles and torsion angles; correlations between esds in cell parameters are only used when they are defined by crystal symmetry. An approximate (isotropic) treatment of cell esds is used for estimating esds involving l.s. planes.

### Fractional atomic coordinates and isotropic or equivalent isotropic displacement parameters (Å^2^)

**Table:**

	*x*	*y*	*z*	*U*_iso_*/*U*_eq_	
C1	0.4002 (6)	0.8359 (3)	0.80585 (12)	0.0711 (8)	
H1	0.274222	0.794587	0.794336	0.085*	
C2	0.4122 (8)	0.8741 (4)	0.85639 (13)	0.0839 (11)	
H2	0.293567	0.858606	0.878430	0.101*	
C3	0.5971 (8)	0.9343 (4)	0.87390 (14)	0.0897 (12)	
H3	0.604483	0.958968	0.907768	0.108*	
C4	0.7710 (7)	0.9581 (4)	0.84151 (14)	0.0877 (11)	
H4	0.896441	0.999348	0.853340	0.105*	
C5	0.7611 (6)	0.9208 (4)	0.79108 (13)	0.0764 (9)	
H5	0.880417	0.937415	0.769367	0.092*	
C6	0.5764 (5)	0.8595 (3)	0.77256 (11)	0.0626 (7)	
C7	0.5717 (5)	0.8175 (3)	0.71772 (11)	0.0642 (8)	
C8	0.6388 (6)	0.9135 (4)	0.67725 (13)	0.0751 (9)	
H8A	0.628167	0.996095	0.689444	0.090*	
H8B	0.783448	0.898561	0.661620	0.090*	
C9	0.4381 (6)	0.8741 (3)	0.64378 (12)	0.0695 (8)	
H9	0.486075	0.814897	0.618184	0.083*	
C10	0.3400 (5)	0.8091 (4)	0.69128 (12)	0.0706 (9)	
H10A	0.223403	0.854686	0.708437	0.085*	
H10B	0.291717	0.726837	0.684630	0.085*	
C11	0.7026 (7)	0.6997 (4)	0.71183 (14)	0.0864 (10)	
H11A	0.698104	0.674197	0.676846	0.130*	
H11B	0.855370	0.712428	0.721945	0.130*	
H11C	0.636589	0.638486	0.732996	0.130*	
C12	0.2935 (6)	0.9681 (3)	0.62006 (11)	0.0664 (8)	
C13	0.3006 (7)	1.0888 (3)	0.62654 (14)	0.0823 (10)	
H13	0.408013	1.128814	0.645944	0.099*	
C14	−0.0010 (6)	1.0208 (3)	0.57345 (11)	0.0636 (8)	
C15	−0.3247 (6)	1.0930 (3)	0.52675 (12)	0.0682 (8)	
C16	−0.5193 (6)	1.0635 (3)	0.49439 (13)	0.0712 (9)	
H16	−0.622658	1.126176	0.491776	0.085*	
C17	−0.5769 (6)	0.9646 (3)	0.46806 (12)	0.0710 (8)	
H17	−0.714462	0.969359	0.451502	0.085*	
C18	−0.4561 (6)	0.8490 (3)	0.46116 (12)	0.0674 (8)	
N1	0.1187 (4)	0.9299 (2)	0.58924 (9)	0.0609 (6)	
N2	−0.1832 (5)	1.0037 (2)	0.54206 (10)	0.0677 (7)	
H2A	−0.209319	0.931474	0.531402	0.081*	
O1	−0.2951 (5)	1.1985 (2)	0.54005 (10)	0.0859 (7)	
O2	−0.3074 (5)	0.8116 (2)	0.48857 (12)	0.0998 (9)	
O3	−0.5286 (4)	0.7906 (2)	0.42168 (8)	0.0776 (7)	
H3A	−0.459781	0.726735	0.418911	0.116*	
S1	0.0856 (2)	1.16026 (8)	0.59435 (4)	0.0829 (3)	

### Atomic displacement parameters (Å^2^)

**Table:**

	*U*^11^	*U*^22^	*U*^33^	*U*^12^	*U*^13^	*U*^23^
C1	0.0678 (18)	0.073 (2)	0.0730 (18)	0.0039 (18)	0.0067 (16)	0.0099 (16)
C2	0.093 (3)	0.088 (3)	0.0708 (19)	0.018 (2)	0.018 (2)	0.0131 (17)
C3	0.104 (3)	0.098 (3)	0.067 (2)	0.025 (3)	−0.006 (2)	−0.0036 (18)
C4	0.082 (2)	0.100 (3)	0.082 (2)	0.007 (2)	−0.016 (2)	−0.012 (2)
C5	0.0613 (18)	0.092 (3)	0.076 (2)	0.0016 (18)	−0.0035 (16)	−0.0028 (17)
C6	0.0600 (16)	0.0635 (19)	0.0644 (15)	0.0079 (16)	−0.0002 (14)	0.0052 (13)
C7	0.0552 (16)	0.070 (2)	0.0676 (16)	−0.0017 (16)	0.0036 (13)	0.0008 (14)
C8	0.0637 (19)	0.095 (3)	0.0663 (18)	−0.0158 (17)	0.0057 (14)	0.0062 (16)
C9	0.0714 (18)	0.075 (2)	0.0626 (16)	−0.0095 (17)	−0.0003 (15)	−0.0063 (14)
C10	0.0590 (17)	0.077 (2)	0.0757 (18)	−0.0103 (16)	−0.0003 (15)	0.0048 (16)
C11	0.076 (2)	0.087 (3)	0.096 (2)	0.012 (2)	0.0016 (19)	−0.016 (2)
C12	0.0735 (19)	0.071 (2)	0.0550 (15)	−0.0107 (17)	0.0018 (15)	−0.0046 (13)
C13	0.095 (3)	0.073 (3)	0.079 (2)	−0.015 (2)	−0.015 (2)	−0.0097 (17)
C14	0.073 (2)	0.058 (2)	0.0592 (15)	−0.0101 (15)	0.0050 (14)	−0.0059 (13)
C15	0.083 (2)	0.0537 (19)	0.0677 (18)	−0.0004 (16)	0.0095 (16)	0.0001 (13)
C16	0.077 (2)	0.060 (2)	0.0762 (19)	0.0093 (16)	−0.0005 (16)	0.0013 (15)
C17	0.0756 (19)	0.061 (2)	0.0759 (18)	0.0136 (17)	−0.0069 (17)	0.0009 (15)
C18	0.0757 (19)	0.0570 (18)	0.0696 (17)	−0.0036 (16)	−0.0085 (16)	−0.0025 (15)
N1	0.0687 (15)	0.0577 (15)	0.0565 (13)	−0.0073 (12)	0.0034 (12)	−0.0068 (11)
N2	0.0778 (16)	0.0501 (14)	0.0753 (15)	−0.0039 (13)	−0.0071 (14)	−0.0072 (12)
O1	0.1089 (18)	0.0548 (14)	0.0940 (15)	−0.0059 (13)	0.0042 (14)	−0.0051 (12)
O2	0.1046 (19)	0.0668 (16)	0.128 (2)	0.0217 (15)	−0.0505 (18)	−0.0262 (14)
O3	0.0922 (16)	0.0679 (14)	0.0727 (13)	0.0082 (12)	−0.0112 (12)	−0.0049 (11)
S1	0.1047 (7)	0.0564 (5)	0.0875 (6)	−0.0127 (5)	−0.0120 (5)	−0.0076 (4)

### Geometric parameters (Å, º)

**Table:**

C1—C6	1.391 (5)	C10—H10B	0.9700
C1—C2	1.393 (5)	C11—H11A	0.9600
C1—H1	0.9300	C11—H11B	0.9600
C2—C3	1.368 (6)	C11—H11C	0.9600
C2—H2	0.9300	C12—C13	1.346 (5)
C3—C4	1.366 (6)	C12—N1	1.386 (4)
C3—H3	0.9300	C13—S1	1.727 (4)
C4—C5	1.386 (5)	C13—H13	0.9300
C4—H4	0.9300	C14—N1	1.301 (4)
C5—C6	1.382 (5)	C14—N2	1.377 (4)
C5—H5	0.9300	C14—S1	1.716 (3)
C6—C7	1.511 (4)	C15—O1	1.230 (4)
C7—C11	1.526 (5)	C15—N2	1.360 (4)
C7—C10	1.550 (4)	C15—C16	1.475 (5)
C7—C8	1.553 (4)	C16—C17	1.338 (5)
C8—C9	1.547 (5)	C16—H16	0.9300
C8—H8A	0.9700	C17—C18	1.479 (5)
C8—H8B	0.9700	C17—H17	0.9300
C9—C12	1.488 (5)	C18—O2	1.215 (4)
C9—C10	1.553 (5)	C18—O3	1.295 (4)
C9—H9	0.9800	N2—H2A	0.8600
C10—H10A	0.9700	O3—H3A	0.8200
			
C6—C1—C2	120.0 (4)	C9—C10—H10A	113.7
C6—C1—H1	120.0	C7—C10—H10B	113.7
C2—C1—H1	120.0	C9—C10—H10B	113.7
C3—C2—C1	120.6 (4)	H10A—C10—H10B	110.9
C3—C2—H2	119.7	C7—C11—H11A	109.5
C1—C2—H2	119.7	C7—C11—H11B	109.5
C4—C3—C2	119.9 (3)	H11A—C11—H11B	109.5
C4—C3—H3	120.1	C7—C11—H11C	109.5
C2—C3—H3	120.1	H11A—C11—H11C	109.5
C3—C4—C5	120.2 (4)	H11B—C11—H11C	109.5
C3—C4—H4	119.9	C13—C12—N1	113.6 (3)
C5—C4—H4	119.9	C13—C12—C9	128.5 (3)
C6—C5—C4	121.0 (3)	N1—C12—C9	117.8 (3)
C6—C5—H5	119.5	C12—C13—S1	111.6 (3)
C4—C5—H5	119.5	C12—C13—H13	124.2
C5—C6—C1	118.3 (3)	S1—C13—H13	124.2
C5—C6—C7	120.0 (3)	N1—C14—N2	121.2 (3)
C1—C6—C7	121.7 (3)	N1—C14—S1	115.3 (2)
C6—C7—C11	110.4 (3)	N2—C14—S1	123.5 (3)
C6—C7—C10	117.4 (3)	O1—C15—N2	121.1 (3)
C11—C7—C10	111.1 (3)	O1—C15—C16	119.0 (3)
C6—C7—C8	115.8 (3)	N2—C15—C16	119.9 (3)
C11—C7—C8	112.5 (3)	C17—C16—C15	132.8 (3)
C10—C7—C8	88.0 (2)	C17—C16—H16	113.6
C9—C8—C7	89.8 (2)	C15—C16—H16	113.6
C9—C8—H8A	113.7	C16—C17—C18	130.1 (3)
C7—C8—H8A	113.7	C16—C17—H17	114.9
C9—C8—H8B	113.7	C18—C17—H17	114.9
C7—C8—H8B	113.7	O2—C18—O3	123.1 (3)
H8A—C8—H8B	110.9	O2—C18—C17	125.4 (3)
C12—C9—C8	119.3 (3)	O3—C18—C17	111.5 (3)
C12—C9—C10	116.1 (3)	C14—N1—C12	111.3 (3)
C8—C9—C10	88.1 (2)	C15—N2—C14	124.5 (3)
C12—C9—H9	110.5	C15—N2—H2A	117.7
C8—C9—H9	110.5	C14—N2—H2A	117.7
C10—C9—H9	110.5	C18—O3—H3A	109.5
C7—C10—C9	89.7 (2)	C14—S1—C13	88.22 (18)
C7—C10—H10A	113.7		
			
C6—C1—C2—C3	−0.4 (6)	C8—C9—C10—C7	−15.9 (3)
C1—C2—C3—C4	0.5 (6)	C8—C9—C12—C13	−5.7 (5)
C2—C3—C4—C5	−0.3 (6)	C10—C9—C12—C13	97.8 (4)
C3—C4—C5—C6	0.1 (6)	C8—C9—C12—N1	178.4 (3)
C4—C5—C6—C1	0.0 (5)	C10—C9—C12—N1	−78.1 (4)
C4—C5—C6—C7	−178.7 (3)	N1—C12—C13—S1	0.4 (4)
C2—C1—C6—C5	0.1 (5)	C9—C12—C13—S1	−175.7 (3)
C2—C1—C6—C7	178.8 (3)	O1—C15—C16—C17	168.3 (4)
C5—C6—C7—C11	80.9 (4)	N2—C15—C16—C17	−12.7 (6)
C1—C6—C7—C11	−97.7 (4)	C15—C16—C17—C18	−2.2 (7)
C5—C6—C7—C10	−150.3 (3)	C16—C17—C18—O2	19.8 (6)
C1—C6—C7—C10	31.1 (5)	C16—C17—C18—O3	−160.4 (4)
C5—C6—C7—C8	−48.3 (4)	N2—C14—N1—C12	179.7 (3)
C1—C6—C7—C8	133.0 (3)	S1—C14—N1—C12	−0.1 (3)
C6—C7—C8—C9	−135.5 (3)	C13—C12—N1—C14	−0.2 (4)
C11—C7—C8—C9	96.2 (3)	C9—C12—N1—C14	176.4 (3)
C10—C7—C8—C9	−15.8 (3)	O1—C15—N2—C14	1.0 (5)
C7—C8—C9—C12	134.9 (3)	C16—C15—N2—C14	−178.0 (3)
C7—C8—C9—C10	15.8 (3)	N1—C14—N2—C15	175.5 (3)
C6—C7—C10—C9	134.0 (3)	S1—C14—N2—C15	−4.7 (4)
C11—C7—C10—C9	−97.6 (3)	N1—C14—S1—C13	0.3 (3)
C8—C7—C10—C9	15.8 (3)	N2—C14—S1—C13	−179.5 (3)
C12—C9—C10—C7	−137.8 (3)	C12—C13—S1—C14	−0.4 (3)

### Hydrogen-bond geometry (Å, º)

**Table:**

*D*—H···*A*	*D*—H	H···*A*	*D*···*A*	*D*—H···*A*
C16—H16···O1^i^	0.93	2.35	3.233 (4)	159
N2—H2*A*···O2	0.86	1.83	2.651 (4)	158
O3—H3*A*···N1^ii^	0.82	1.81	2.607 (3)	165

Symmetry codes: (i) *x*−1/2, −*y*+5/2, −*z*+1; (ii) *x*−1/2, −*y*+3/2, −*z*+1.
